# Machine Learning-Based Air-to-Ground Channel Model Selection Method for UAV Communications Using Digital Surface Model Data

**DOI:** 10.3390/s22239234

**Published:** 2022-11-27

**Authors:** Young-Eun Kang, Young-Ho Jung

**Affiliations:** School of Electronics and Information Engineering, Korea Aerospace University, Goyang 10540, Republic of Korea

**Keywords:** channel model selection, UAV communication, machine learning, air-to-ground (A2G) channel model, UAM, digital surface model, deep neural network (DNN), convolutional neural network (CNN)

## Abstract

This paper proposes an automatic air-to-ground (A2G) channel model selection method based on machine learning (ML) using digital surface model (DSM) terrain data. In order to verify whether a communication network for a new non-terrestrial user service such as Urban Air Mobility (UAM) satisfies the required performance, it is necessary to perform a simulation reflecting the characteristics of the corresponding terrain environments as accurately as possible. For this simulation, A2G channel models corresponding to various terrain environments and a method of automatically classifying the terrain type of the simulation area must be provided. Many A2G channel models based on actual measurement results exist, but the practical automatic topography classification method still needs to be developed. This paper proposes the first practical automatic topography classification method using a two-step neural network-based classifier utilizing various geographic feature data as input. Since there is no open topography dataset to evaluate the accuracy of the proposed method, we built a new dataset for five topography classes that reflect the characteristics of Korea’s topography, which is also a contribution of our study. The simulation results using the new data set show that the proposed ML-based method could increase the selection accuracy compared to the technique for direct classification by humans or the existing cross-correlation-based classification method. Since the proposed method utilizes the DSM data, open to the public, it can easily reflect the different terrain characteristics of each country. Therefore, the proposed method can be effectively used in the realistic performance evaluation of new non-terrestrial communication networks utilizing vast airspace such as UAM or 6G mobile communications.

## 1. Introduction

The research and industrialization of wireless communication for the past decades have mainly focused on providing a communication link for users on the ground with high speed and reliability. Recently, the industry’s interest in drones and Urban Air Mobility (UAM) has increased. So various research and standardization activities are carried out to secure a stable communication link for air vehicles such as Unmanned Aerial Vehicles (UAVs) and vertical takeoff and landing (VTOL). Non-terrestrial air networks can be classified based on the service altitude. Unmanned Aircraft System (UAS) Traffic Management (UTM) system provides automatic control service using existing 4G and 5G terrestrial networks to safely operate drones in airspace below 150 m [1,2]. The UAM system provides passenger transportation services such as air taxis in the airspace of 300 m to 600 m [3,4]. For UAM, the service is initially started based on terrestrial mobile communication networks such as 4G and 5G, and low-orbit satellite networks or the Control and Non-Payload Communication (CNPC) [5] system for the unmanned air vehicle (UAV) will be additionally used in the future [4]. In addition, the 3rd Generation Partnership Project (3GPP) is actively carrying out standardization activities for efficient coverage expansion of three-dimensional airspace [6,7,8,9]. In order to extend the coverage of the cellular network to the sky, there are many technical issues, such as uplink and downlink interference management [6,7] and identification of users in the air [7]. The impact on the system structure of the 5G network for UAV identification and control [8] and the application of the 5G network for UTM application is also studied [9].

For research and standardization of new communication systems in the airspace, it is essential to simulate communication performance using a channel model that reflects the actual propagation characteristics of signals between UAVs and ground stations. Therefore, various efforts have been made to study the channel model based on the air-to-ground (A2G) measurement results in various terrain environments. The selection of appropriate channel measurement parameters in each environment is vital to obtain accurate channel statistics. The A2G propagation environment is generally classified into flat land, hills, mountains, and water surfaces, and in particular, the ground has additional characteristics such as grass, forests, and buildings. In this regard, the International Telecommunication Union (ITU) classified the popular topography classes and provided them as a list [10]. Based on this, in [11], the measurement environment and A2G propagation scenario were organized by the topographical environments, and the difference in channel characteristics according to the different terrains was specified. Various studies have been conducted to develop A2G channel models in several types of topography, such as water surfaces [12,13,14], mountains [15], cities [16,17], and suburbs [16], based on actual measurement results using airplanes or UAVs. As a result, parameters such as pass loss, delay spread, K-factor, and the first and second-order statistics in the various frequency bands are different for each topography class. Based on the measurement results, a channel model that can be used in the simulation can be created. A stochastic model can be created based on measurement parameters [14,15,16,18], or a deterministic channel model can be created in combination with ray tracing [19].

For evaluating the performance of the communication systems in the air domain, it is necessary to select and reflect a channel model suitable for the area to be simulated among the channel models for each terrain environment. The task to be solved in this paper is automatically identifying the topography type of the performance simulation target area by using digital topographical information. Based on this, a channel model corresponding to the simulation area is automatically selected and used to simulate communication system performance. There are several types of topographical information, such as the Digital Elevation Model (DEM) and the Digital Surface Model (DSM) [20]. However, we use DSM in this paper because only DSM reflects ground structures such as buildings and trees, and it is open to the public [21].

An appropriate channel model for a specific region can be determined by human intuition based on the information known in advance on the region. However, it is very cumbersome when analyzing the network capacity for a wide area corresponding to a specific state or country through repeated simulations. Although it is possible to simulate communication performance in a small area, it is very cumbersome when analyzing the network capacity for a wide area corresponding to a specific state or country through repeated simulations. For example, to develop the communication network for UAM, it is necessary to simulate a wide area of airways corresponding to a distance of more than hundreds of kilometers. For another example, to optimize the ground stations’ position by simulating the UAV command and control network coverage such as CNPC, it is necessary to select a channel model suitable for each detailed area’s topography and apply it to the simulation. If a simulation is performed by selecting a channel model suitable for the topography of the corresponding area for each 10 square-kilometer area, about 10,000 selections are required for South Korea, and about 42,000 selections are necessary for the state of California. A person can’t decide one by one, and a method for automatically selecting a channel model suitable for each experimental topography among multiple channel models is essential. For the automatic channel model selection method, there is a method using the correlation coefficient between the test area’s altitude probability mass function (PMF) and the altitude PMF of the region where the actual measurement was performed for each channel model [22]. Even with the same topography, the absolute altitude varies significantly from country to country. Therefore, in [22], instead of the absolute-altitude PMF, the relative-altitude PMF, calculated for a constant number of steps between the maximum and minimum altitudes of each test area, was used for calculating the correlation values. In addition, it was shown that the geographic area of Korea could be well mapped with the topographical environments in which the channel models were developed. However, only a limited number of examples were presented to show the possibility of application. Except for the results in [22], there is no research result on the automatic topographic classification method.

Machine learning (ML)technology is widely used in various academic fields, and its applicability to UAV communication is also being studied [23,24]. In this paper, we proposed a method of classifying the topography using the well-known convolutional neural network (CNN) and deep neural network (DNN) methods using DSM data from various regions in Korea. In particular, we propose a two-step neural network (NN) method in which the classification process consists of successive steps utilizing two separate NN models to improve classification performance. Suppose the classification error is significant in the specific topography classes in the first step. Then, the corresponding classification classes are set as one combined class, and the primary classification is performed. Then if the first classification result is the combined class, the second classification is performed among the confused classes using a separate neural network. The main idea is to enhance the accuracy by classifying the classes with high mutual confusion in the second step using a different classifier.

There are two problems in the performance evaluation of the proposed method. One is that there is no terrain data set for testing, and the other problem is that there is only one existing method for comparison [22]. To overcome the problems, we set five types of topography classes reflecting the topographical characteristics of the Korean Peninsula and generat a total of 1000 data, 200 for each category based on the known information for the region of data. Using this data, we compare the classification performance through experiments applying various classification methods. In order to compensate for the fact that there is only one reference for performance comparison, we also conducted human classification from the DSM picture. As a result of the experiment, it is confirmed that the accuracy of the proposed two-step NN-based classification method increases by 20%p compared to the existing correlation method in [22], and there is a performance improvement of 11.2%p compared to the human classification from the DSM picture.

The contributions of this paper are as follows:

- We build a new topography dataset for five types of terrain that can be used for automatic topography classification problems.
- Using ML, we propose a practical classifier structure that automatically classifies the terrain type of the analysis region.
- A geospatial information processing method that can be used when classifying terrain is presented, and feature values with excellent performance are analyzed.
- We propose a two-step NN-based classifier, which can significantly reduce the classification error.
- In conclusion, we propose the first practical automatic terrain type classification method that can be used for the performance evaluation of new communication systems for airspace in a wide range of simulation regions reflecting the actual environment.

The structure of the paper is as follows. Section 2 describes the A2G channel model according to the topography and the construction of a dataset for the automatic topographic classification performance test. Section 3 describes the feature extraction methods for applying ML techniques, and Section 4 describes the proposed ML-based automated channel model selection methods. Section 5 presents the simulation environment and results for comparing the classification performance of the proposed and existing methods, and Section 6 concludes the paper.

## 2. Background of Research and Construction of New Dataset

### 2.1. Summary of Existing Research on A2G Channel Models by Terrain

**Figure 1:**
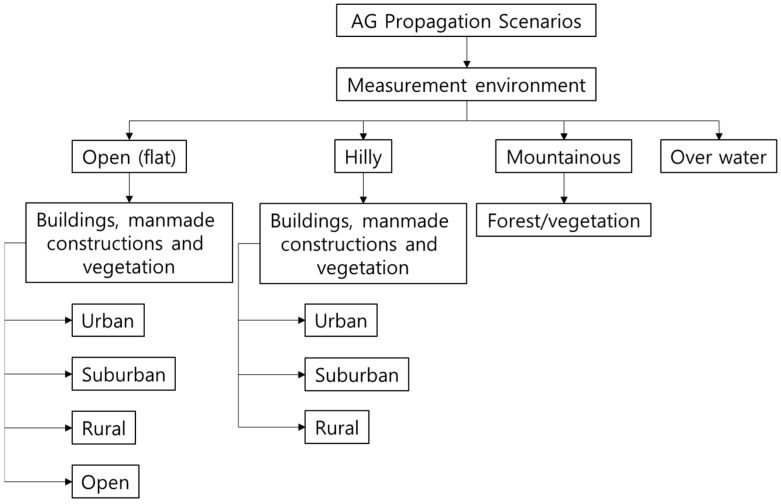
Classification of A2G propagation topography [11].

Various research results have been derived recently for studying A2G channel models in the air domain [11,12,13,14,15,16,17]. Among them, representative results measured channel characteristics by mounting channel-sounding equipment on an aircraft and derived channel models based on the measured data. Figure 1 shows the classification of channel measurement environments according to the terrain. It can be broadly divided into flat land, hill, mountain, and water. Flat land and hill area can be subdivided into downtown, suburban, and rural area, and water areas can be divided into the sea and freshwater. Table 1 summarizes the contents of A2G channel model studies conducted through aerial measurements in various literature. The criteria for categorizing the topographic environment are not absolute because the average altitude, mountain height, buildings, etc., vary depending on the country. Therefore, the number of classification classes can differ for different countries.

### 2.2. Building a Dataset for the Topography of South Korea

**Figure 2:**
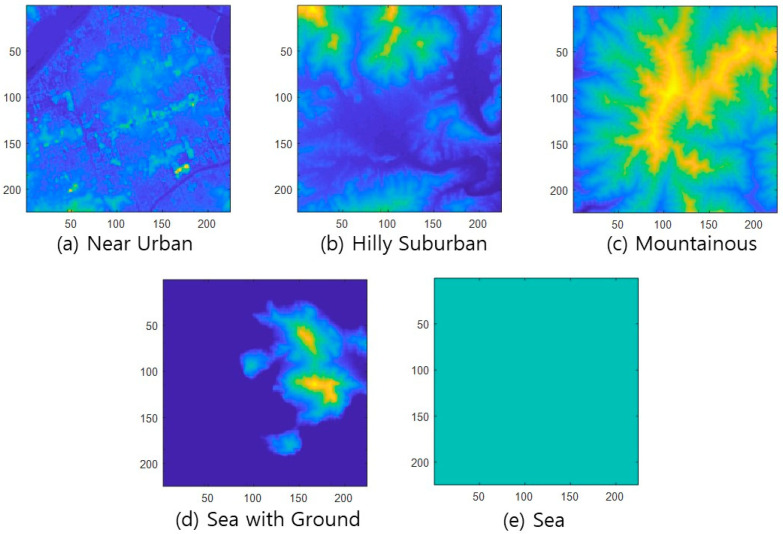
DSM example of Korean topographic classes.

This paper aims to classify Korean topography according to the characteristics of Korean topography and to derive a method for automatic classification by applying ML techniques using topographic information data processed in various forms. Since there is no publicly available data set for topography type classification, we construct a new dataset using 30 m resolution DSM for the entire area of South Korea. In this paper, we categorize the topography of Korea into five types: *Near Urban*, *Hilly Suburban*, *Mountainous*, *Sea with the Ground*, and *Sea*. Compared to the topography of the United States, the Korean Peninsula is small in area and has a low degree of diversity in topography. For example, the topography of the United States has terrain such as deserts, and the steepness of mountains varies greatly, but this is not the case in Korea. So, it is efficient and sufficient to classify Korean topography into five categories. Table 2 provides a brief description of the five topography classes. *Near Urban* refers to urban topography and is defined as an area with many buildings. *Hilly Suburban* is a suburban terrain and includes mainly small hills or low buildings with trees, forests, and rice fields. *Mountainous* is a mountainous area. *Sea with the Ground* refers to an area with both land and sea as the coast. *Sea* is a terrain of only water with zero altitudes. Figure 2 shows an example of five types of topography using DSM data with a resolution of 30 m.

In this paper, the terrain feature data used for applying ML is the altitude information at the longitude and latitude of each grid point in the analysis area. There are two types of altitude information. First, there is a DEM. The DEM is a topographic model that expresses the bare earth part of topographic information, excluding buildings, trees, and artificial structures. Second, DSM is a model representing all information in the real world, such as trees, buildings, and artificial structures [19]. Since buildings and artificial structures are essential in classifying urban and suburban environments, we use DSM in this paper. We construct the Korean topography dataset by selecting 200 data samples for each topography class, checking the satellite images from all over South Korea, and labeling them. Each image covers a three km-by- three km area.

## 3. Topographic Feature Extraction Methods for ML Application

### 3.1. Input Data Processing Methods for Applying DNN

The number of input nodes is equal to the length of the input vector, and the number of output nodes is the number of classes to be identified. Since the altitude information is too large to put itself as an input value of the DNN, the PMF value of the altitude information is extracted and used in the DNN as an input feature value. Both absolute and relative altitudes can be the input of the DNN. If the number of input nodes is *N*, to obtain the absolute-altitude PMF value, divide the altitude range from 0 to the preset maximum height value into *N* ranges and calculate the probability value of the corresponding altitude range. Since the minimum altitude value is 0 m when the highest altitude value is $h_{max}$, the interval for each altitude section is $\Delta_{A}=h_{max}/N$, and the probability value corresponding to the *n*-th altitude section according to the absolute-altitude PMF, $p_{A}\left(n\right),$ is expressed as Equation (1), where P[·] is the probability function.

(1) $$p_{A}\left(n\right)=P\left[\left(n-1\right)\Delta_{A}\leq altitude\leq n\Delta_{A}\right],\;\;\;\;\;\;\;1\leq n\leq N.$$

Since there is a significant difference in the average elevation and the elevation difference by topography in each country, the elevation difference in the analysis area may be more important than the absolute elevation value. If $h_{min,R}$ is the lowest altitude, and $h_{max,R}$ is the highest altitude within the terrain type analysis area, the interval between each altitude section is $\Delta_{R}=\left(h_{max,R}-h_{min,R}\right)/N$, and the probability value corresponding to the *n*-th altitude section according to the relative-altitude PMF, $p_{R}\left(n\right),$ is expressed as:

(2) $$p_{R}\left(n\right)=P\left[h_{min,R}+\left(n-1\right)\Delta_{R}\leq altitude\leq h_{min,R}+n\Delta_{R}\right],\;\;\;\;\;\;\;\;1\leq n\leq N.$$

If the absolute and relative-altitude PMFs are used simultaneously, after obtaining each PMF of *N*/2 ranges, we can combine them as *N* values and apply them as the input of the DNN.

### 3.2. Input Data Processing Methods for Applying CNN

**Figure 3:**
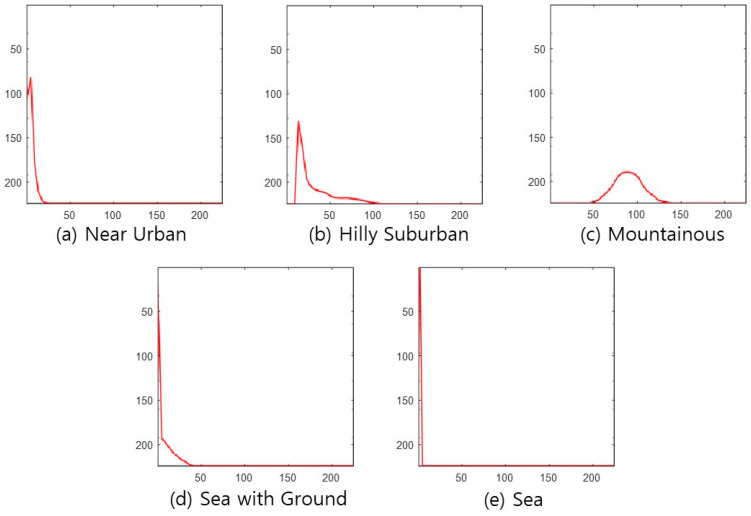
Example of absolute-altitude PMF for each topography class.

**Figure 4:**
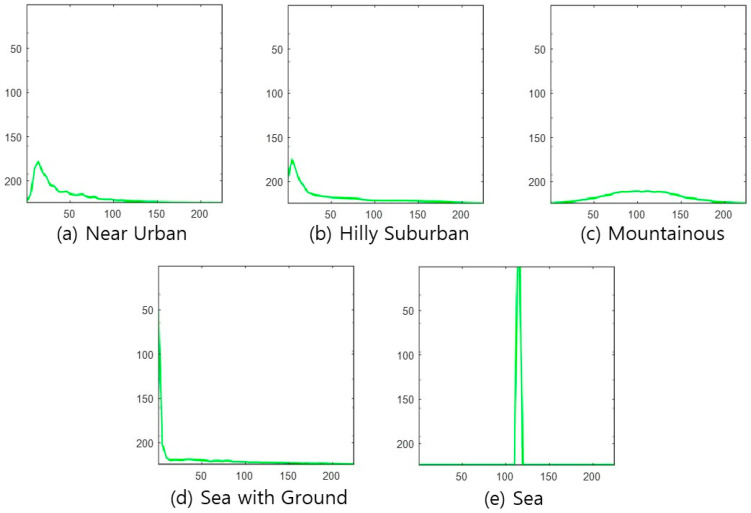
Example of relative-altitude PMF for each topography class.

**Figure 5:**
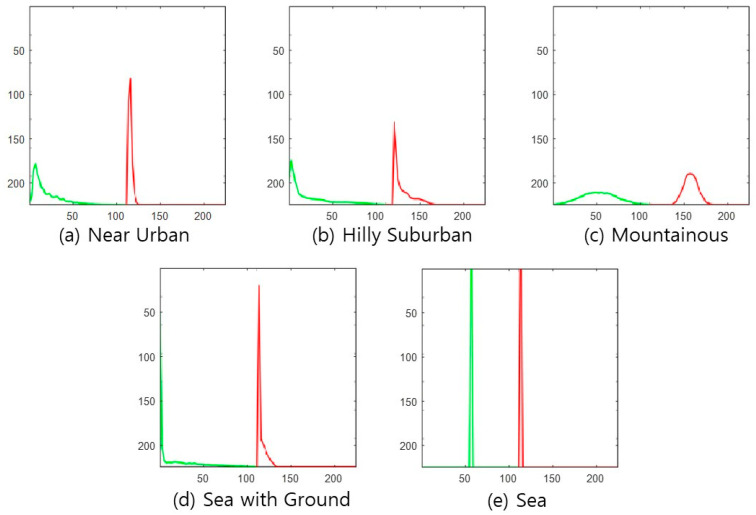
Example of a combination of absolute and relative-altitude PMF for each topography class.

CNN receives a Red-Greed-Blue (RGB) color image as an input, goes through several convolutional layers, and outputs the identification result at the output node corresponding to each candidate to be identified. This paper uses the following four types of images, which reflect each topography class’s characteristics, as CNN input images. The images in Figure 2, Figure 3, Figure 4 and Figure 5 are used as CNN inputs. Each image is 224-by-224 pixels, and when used as CNN inputs, horizontal and vertical axis legends expressing the pixel numbers and borders are removed before use.

- DSM figure—The first feature is the DSM picture, as shown in Figure 2.
- Absolute-altitude PMF—The second is a graph of the absolute-altitude PMF value used in the DNN, as shown in Figure 3. *Near Urban* and *Hilly Suburban* are similar in the picture, and *Sea with the Ground* and *Sea* also seem similar.
- Relative-altitude PMF—The third is the relative-altitude PMF figure, as shown in Figure 4, and the horizontal axis of each figure represents different altitude values. Compared with the absolute-altitude PMF in Figure 3, the distinction between *Sea with the Ground* and *Sea* is clear, whereas the *Near Urban* and *Hilly Suburban* cases look almost the same.
- Combination of Absolute and Relative-altitude PMF—Fourth, to consider both the relative-altitude PMF and the absolute-altitude PMF, the two graphs are serially plotted, as shown in Figure 5. When both features are considered together, improvement in accuracy is expected. To distinguish two PMFs, the relative-altitude PMF is indicated in green, and the absolute-altitude PMF is displayed in red.

## 4. Proposed ML Models

In this paper, we applied various machine learning methods using the input signals for DNN and CNN presented in Section 2. The machine learning structure, DNN, and CNN implementation methods will be explained in this section, and the performance evaluation results will be presented in the next section.

### 4.1. Structure of the Proposed ML-Based Automatic Topography Class Classifier

**Figure 6:**
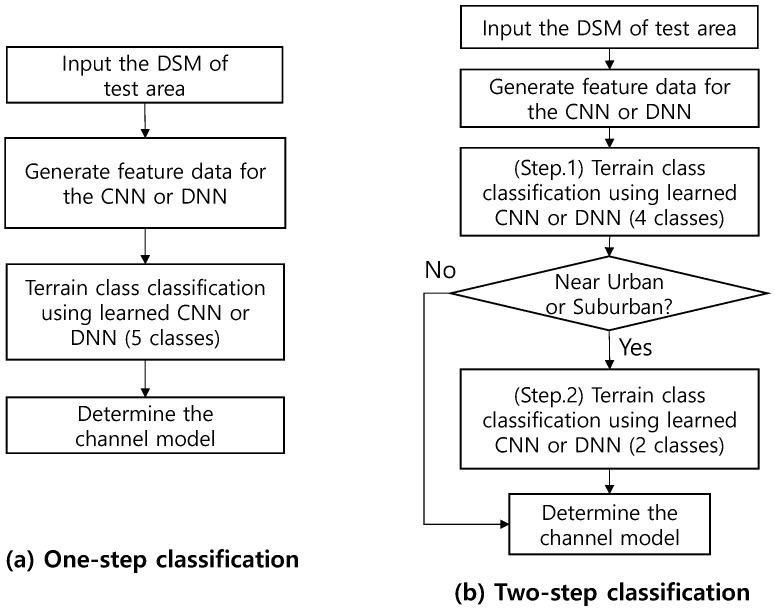
The decision process of the proposed NN-based topography class classification.

In this paper, as shown in Figure 6, we implement two types of learning classifiers and evaluate the classification performance. Figure 6a’s structure is the same as a general ML-based classifier, where one training model is created and determined as one of all candidates for classification. All candidate classes may be classified well with the one-step classifier structure shown in Figure 6a, but there may be situations in which detection errors occur significantly between specific classes. Although detailed simulation results will be presented in Section 5, in the case of the five topographic classification problems dealt with in this paper, when the one-step classifier of Figure 6a is applied, there is a significant classification error between *Near Urban* and *Hilly Suburban*.

The structure to supplement this is the proposed two-stage classifier structure, as shown in Figure 6b. In the two-step classification method in Figure 6b, in the first step, *Near Urban* and *Hilly Suburban*, which cause a lot of mutual classification errors, are combined into one class. Then, the first step is classifying four candidate classes using the classifier trained for *Near Urban & Hilly Suburban*, *Mountainous*, *Sea with the Ground*, and *Sea*. Suppose the first-step classification result is the Near Urban & Hilly Suburban environment. In that case, the second step reclassifies using a NN model trained only on *Near Urban* and *Hilly Suburban*. In the proposed two-step classifier structure, the structure of the NN at each level can be either CNN or DNN, and four different combinations are possible. Therefore, we should find the optimum combination to maximize classification probability through accuracy comparison.

### 4.2. The Structure of DNN and CNN Used for Performance Evaluation

As shown in Table 3, the DNN structure used in the performance evaluation of this paper receives 100 feature values as input, passes through 3 hidden layers, and outputs the results to the output nodes (2, 4, or 5). In the CNN method, it is vital to configure layers that significantly affect performance. This paper uses the Alexnet [25] configuration, which is relatively simple but shows excellent classification performance among CNN models.

## 5. Simulation Method and Results

### 5.1. Simulation Method

The data used in the simulation are all the same. We use the newly constructed 1000 Korean topography data, composed of 200 data for each of the five types of topography classes described in Section 2.2. The classification performance of single-step DNN or CNN-based classifiers and two-step NN classifiers are examined. In the case of a two-stage ML classifier, since either CNN or DNN can be used for stage 1 and stage 2, respectively, we test all four combinations. Table 4 shows the simulation parameters of DNN. Five-fold cross-validation is performed by dividing the total 1000 data into 800 training data and 200 test data, and the number of epochs is set to 200, considering the learning error curve. Table 5 shows CNN-related parameters applied to the experiment. As with DNN, five-fold cross-validation is performed by dividing the total number of 1000 data into 800 training data and 200 test data.

In addition, for performance comparison with the proposed ML-based classifier, we present the experimental results of a correlator-based classifier [22] and a method in which persons directly classify topography.

### 5.2. The Performance Evaluation Results of the NN-Based One-Step Classifier

#### 5.2.1. DNN

**Figure 7:**
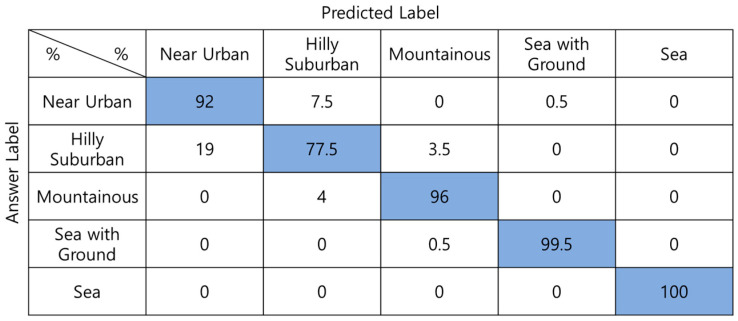
Confusion matrix of DNN-based one-step classification.

Figure 7 shows the confusion matrix of the DNN with 100 input values. Fifty absolute-altitude PMF values and fifty relative-altitude PMF values are used as the input signals, and both types of altitude information are used. The average classification accuracy is 93.0%. *Hilly Suburban* is misclassified as *Near Urban* in many cases.

#### 5.2.2. CNN

**Figure 8:**
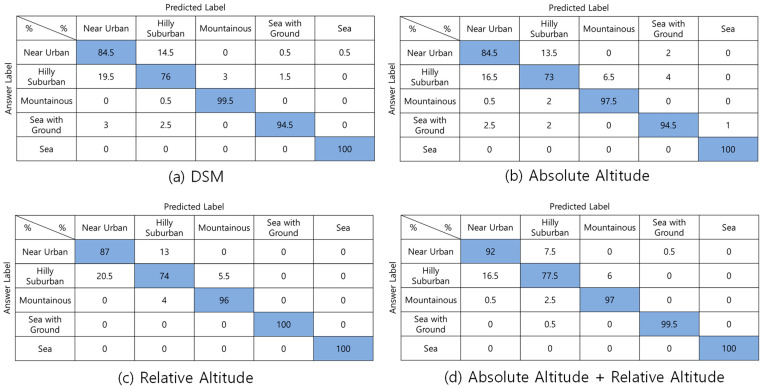
Confusion matrixes of CNN-based one-step classification according to the input features.

Figure 8 compares the classification performance according to the kinds of input data of CNN in 3.1.2. According to the type of input data, classification accuracies are (1) DSM—90.9%; (2) Absolute-altitude—89.9%; (3) Relative-altitude—91.4%; and (4) Absolute-altitude + relative-altitude—93.2%, correspondingly. Among them, the superimposition of absolute and relative-altitude PMF shows the highest accuracy. Such as DNN, *Hilly Suburban* is misclassified as *Near Urban* in many cases.

### 5.3. The Performance Evaluation Results of the NN-Based Two-Step Classifier

**Figure 9:**
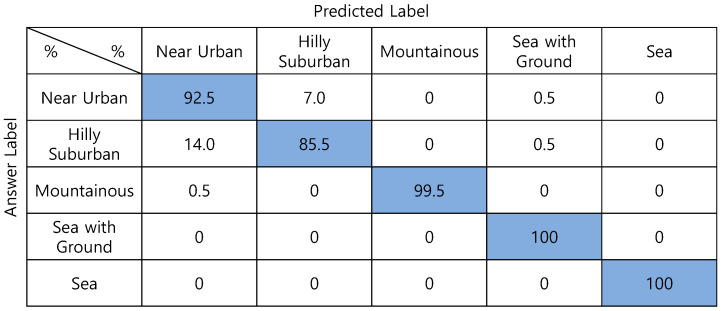
Confusion matrix of the two-step classification (DNN for the 1st step and CNN for the 2nd step).

In the case of a one-step classifier, there were many identification errors between *Near Urban* and *Hilly Suburban*, and to resolve the problem, we propose the two-step classifier. In the first step, one of the *Near Urban & Hilly Suburban*, *Mountainous*, *Sea with the Ground* and *Sea* environments is identified. Then, suppose the first classification result is *Near Urban & Hilly Suburban*, using the second classifier trained on *Near Urban* and *Hilly Suburban*. In that case, it is finally classified into one of them. DNN and CNN can be used for steps 1 and 2, respectively, and the average classification accuracy according to them is shown in Table 6. In the case of the first step, the DNN is superior to CNN by 1.7%p, and in the case of the second step, the CNN showed better identification performance than the DNN by 4.3%p. So, the best combination is DNN + CNN, and the final identification accuracy after two steps is 95.5%, as shown in Figure 9. The reason why the accuracy of the two-step classification increases compared to the one-step classification in 5.2 is as follows. In general, classifier accuracy increases as the number of classification class candidates decreases, and the case of this paper shows the same tendency. According to Figure 8d, when classifying into one of the five classes, 6% of *Hilly Suburban* is incorrectly classified as *Mountainous*, and 3% of *Mountainous* is incorrectly classified as near urban or *Hilly Suburban*. In the case of classifying the four candidates by integrating *Near Urban* and *Hilly Suburban* into one class, the errors are significantly reduced to 0% and 0.5%, respectively. Accuracy can be improved by classifying only the *Near Urban* and *Hilly Suburban* in step 2, so in the case of *Hilly Suburban*, which has the lowest success rate in one-step classification, an 8.0%p improvement in accuracy is obtained from 77.5% to 85.5%.

### 5.4. Simulations of Existing Methods for Performance Comparison

#### 5.4.1. Conventional Correlation-Based Method

**Figure 10:**
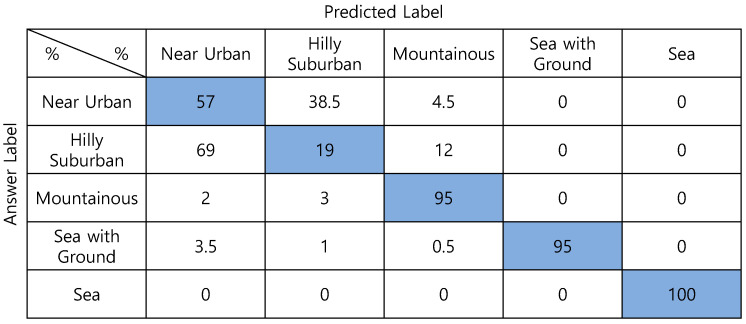
Confusion matrix of the correlation-based classification in [22].

To verify the proposed methods’ superiority, we evaluate the accuracy of the relative-altitude PMF-based cross-correlation method [22]. Since the correlation method does not have a training process, all data are used as test data, and the number of relative-altitude PMF levels is set to 50. The reference PMF for each class is obtained by averaging the relative-altitude PMF of 200 samples. As a result of testing 200 for each class, the average classification accuracy is 73.2%, as shown in Figure 10. Since classification is based only on the cross-correlation value between the representative PMF value corresponding to each topography class candidate and the relative altitude PMF of the test region, the performance degradation compared to the proposed NN-based classification method is significant. In particular, the miss-classification error significantly increases between Near Urban and Hilly Suburban, which have similar PMF shapes shown in Figure 4.

#### 5.4.2. Classified by Human

**Figure 11:**
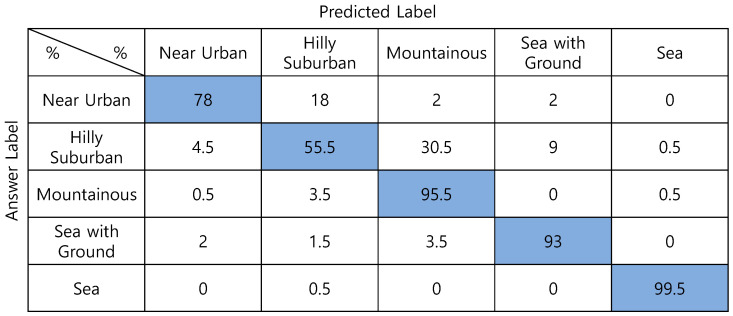
Confusion matrix of classification by a human.

As another performance comparison criterion, humans directly classify the topography by looking at the DSM picture. For learning the features of each topography class, we show feature descriptions of topography classes shown in Table 2 and 50 DSM samples such as Figure 2, corresponding to each class. After that, 100 DSM data for each class are directly classified by seven people. The results are shown in Figure 11, and the average classification accuracy is 84.3%. Unlike the proposed NN-based classification, *Hilly Suburban* is misclassified as *Mountainous* in many cases (30.5%).

### 5.5. Analysis of Experimental Results

Table 7 shows the summary of the simulation results. The proposed ML-based automatic classification method provides significantly better classification accuracy than the existing methods. The proposed two-step classifier using DNN in the first step and CNN in the second step shows the best classification accuracy with 95.5%. The one-step classifier using CNN and DNN also provides excellent accuracy of 93.3% and 93.0%, respectively. Instead of classifying five topography classes at once, an additional 2.3%p improvement in accuracy is obtained through the proposed two-step classification by separating two classes where mutual confusion frequently occurs. The conventional correlation-based method has 22.3%p lower accuracy than the proposed method, and the human classification also shows 11.2%p lower accuracy than the proposed method. Therefore, it is confirmed that the NN-based ML technology can be effectively used for automatic topography classification.

## 6. Conclusions

This paper proposed an automatic A2G channel model selection method using ML. The proposed two-level neural network structure improves classification accuracy by separately classifying two topographical classes, which have many classification errors in a single classifier. The proposed automatic classifier significantly improved the identification performance compared to the existing correlation value-based method or human classification. The limitation of our work is considering only the terrain of South Korea. However, the proposed method in this paper can be easily applied to different countries if only a topographic data set is built for each country. Because DSM data is open to the public, we believe the topography data set for the different countries can be easily constructed based on the information in this paper. So, it is expected that the proposed automatic topography type classification method can be efficiently used to simulate communication performance in various application services such as UTM and UAM utilizing the airspace.

## Figures and Tables

**Table 1:** Examples of A2G channel measurement environments [14,15,16].

Setting	Location	Specific Environment
Over Sea [14]	Oxnard, CA	Open salt water with few stationary structures & watercraft
Urban [16]	Cleveland, OH	Cityscape view with many tall buildings on flat terrain, adjoining open freshwater
Hilly Suburban [16]	Latrobe, PA	A mix of rural terrain & urban structures in the valley, viewed from the airport
Hilly [15]	Latrobe, PA	Ground station (GS) antenna beam to a mountain ridge w/natural cover. Ridge extends into line-of-sight between UAV & GS
Mountainous [15]	Telluride, CO	Very ‘Mountainous’ terrain
Hilly [15]	Palmdale, CA	Dry, hilly terrain with natural cover
Hilly Suburban [16]	Palmdale, CA	Open, flat desert & agricultural terrain
Fresh Water [14]	Cleveland, OH	Open fresh water
Suburban [16]	Cleveland, OH	Suburban, some over-water, flat terrain

**Table 2:** Topography classes in South Korea.

Label	Description
Near Urban	Terrain with artificial buildings and skyscrapers
Hilly Suburban	Suburban terrain, including hills
Mountainous	High-altitude mountainous terrain
Sea with the Ground	Terrain including sea with land or small islands
Sea	Terrain containing only the sea

**Table 3:** Structure of DNN for performance evaluation.

DNN Parameters	Value
Number of input nodes	100
Number of hidden layers	3
Number of nodes for the 1st hidden layer	500
Number of nodes for the 2nd hidden layer	200
Number of nodes for the 3rd hidden layer	40
Number of output nodes	2, 4, 5
Activation function of hidden layers	Sigmoid
Activation function of the output layer	Softmax
Cost function	Cross Entropy
Dropout	0.2
Training method	Stochastic Gradient Descent (SGD)

**Table 4:** Parameters for DNN simulation.

Parameters for DNN	Value
Number of training data	800
Number of test data	200
Epoch number	200
Learning rate	0.001
Momentum	0.001
Mini-batch size	128

**Table 5:** Parameters for CNN simulation.

Parameters for CNN	Value
Number of training data	800
Number of test data	200
Maximum number of Epoch	200
Learning rate	0.01
Training method	SGDM (Stochastic Gradient Descent with Momentum)
Momentum	0.9
Mini-batch size	128

**Table 6:** Classification accuracy of two-step classifier according to types of NN.

	NN	DNN	CNN
Step			
1st step (4 candidates)		99.7%	98.0%
2nd step (2 candidates)		83.5%	87.8%

**Table 7:** Comparison of Classification Accuracy.

Classification Method	Classification Accuracy (%)
Two-step NN (proposed)	95.5
One-step CNN (proposed)	93.2
One-step DNN (proposed)	93.0
Cross-correlation [22]	73.2
Classified by human	84.3

## Data Availability

The data presented in this study are available on request from the corresponding author with restrictions. The data are not publicly available due to the policy of the institute.
